# Hereditary Angioedema with Normal C1 Esterase Inhibitor Refractory to Long-Term Prophylaxis: A Case Report

**DOI:** 10.7759/cureus.33800

**Published:** 2023-01-15

**Authors:** Gavin Cobb, Celina C Bernabe

**Affiliations:** 1 College of Medicine, Kansas City University, Kansas City, USA; 2 Allergy and Immunology, Allergy & Asthma Care PA, Overland Park, USA

**Keywords:** c1 esterase inhibitor, hae with normal c1 inhibitor, hereditary angioedema, covid 19 vaccine, angioedema, hae type iii, hereditary angioedema without normal c1 inhibitor, allergy and immunology, hae

## Abstract

Hereditary angioedema (HAE) was classically thought to be related to a deficiency in the C1 esterase inhibitor (C1-INH). However, HAE with a normal C1 esterase inhibitor (HAE nC1-INH) is a rare disease that has been recently characterized. In this case, we describe a woman with a 21-year history of angioedema who, in the last two years, has experienced severe exacerbations that led to the formal diagnosis of HAE nC1-INH. The patient has been treated with current HAE long-term prophylaxis (LTP) and on-demand therapies but is still experiencing severe, frequent attacks. Our case describes the timeline of this patient’s presentation and provides valuable insight into the presentation and management of HAE nC1-INH.

## Introduction

Hereditary angioedema (HAE) is a rare disease process that is associated with life-threatening sequelae, such as asphyxiation due to edema of the airway, as well as reductions in quality of life for the patient. This disease process has been classically thought of as a deficiency in C1 esterase inhibitor (C1-INH); however, studies have elucidated multiple forms of this disease. HAE with decreased or absent C1 esterase inhibitor (HAE C1-INH), or HAE type 1, and HAE with dysfunctional C1 esterase inhibitor, also known as HAE type 2, have a prevalence estimated to be between 1.1 and 1.6 per 100,000, while HAE nC1-INH is thought to be even rarer [1]. Although uncommon, HAE nC1-INH symptoms are similar to those of HAE C1-INH, including swelling of the face, hands, feet, and airway, as well as infrequent abdominal involvement [2].

Interestingly, several key differences distinguish HAE nC1-INH. HAE nC1-INH is commonly associated with facial swelling [2]. HAE nC1-INH has been described more frequently in women than men and for years was thought to be estrogen-dependent, but it has been recently confirmed in the male population [3]. There have been several genetic mutations linked with HAE nC1-INH in factor XII, plasminogen, angiopoietin, and kininogen-1 genes [4-6]. Due to the ongoing exploration of the genetic component of HAE nC1-INH, the disease is currently diagnosed with a combination of clinical criteria and available genetic markers [7]. The current standard of diagnosis for HAE nC1-INH includes a history of angioedema independent of urticaria, as well as a normal C4 level, C1-INH antigen, and C1-INH function. Additional criteria include either a known genetic mutation associated with the disease or a positive family history of recurrent angioedema that does not respond to high-dose antihistamine therapy [7]. We present a case of HAE nC1-INH refractory to initial rounds of standard-of-care treatment.

## Case presentation

A 38-year-old female presented to an outpatient allergy clinic with a history of angioedema beginning at age 16. Her family medical history was pertinent for idiopathic angioedema in both her mother and grandmother, unfortunately leading to the passing of her mother. The patient has a history of angioedema flares that she attributes to taking angiotensin receptor blockers, receiving lidocaine injections, and eating mango. In 2020, the patient received her first vaccine for SARS-CoV-2, Spikevax (COVID-19 mRNA vaccine), which was followed by an anaphylactic reaction and required admission to the intensive care unit (ICU). Following that incident, the patient experienced frequent, severe attacks that prompted her first consultation with an outpatient allergy clinic. The working diagnosis of HAE nC1-INH was entertained as her C1 esterase inhibitor level, C1 esterase inhibitor function, and C1q were normal. Table 1 presents the full panel of laboratory tests.

**Table 1:** Hereditary angioedema laboratory panel

Test	Result	Normal range
Complement C4	42 mg/dl	10-49 mg/dl
Complement C3	149 mg/dl	88-200 mg/dl
Complement C1q	19	12-22 mg/dl
C1 esterase inhibitor function	>91%	>67%
C1 esterase inhibitor	41 mg/dl	19-37 mg/dl
Galactose-alpha-1,3-galactose IgE	<0.10 kU/L	<0.10 kU/L

The patient was also screened genetically via the Invitae Hereditary Angioedema Panel, and no pathogenic mutations were found. Results are shown in Table 2.

**Table 2:** Invitae hereditary angioedema panel ANGPT1: Angiopoietin 1; F12: Coagulation Factor XII; SERPING1: Serpin Family G Member 1; PLG: Plasminogen

Gene	Transcript	Result
ANGPT1	NM_001146.4	No pathogenic variant found
F12	NM_000505.3	No pathogenic variant found
SERPING1	NM_000062.2	No Pathogenic variant found
PLG	NM_000301.3	No pathogenic variant found

The patient was then referred to our allergy clinic, and on initial presentation, the physical exam was positive for hoarseness, right upper lid swelling with visual impairment, and tongue swelling. At that time, acute (on-demand) therapy, Ruconest (an intravenous recombinant C1 esterase inhibitor), was administered, and within 30 to 45 minutes, her symptoms dramatically improved by 75 to 80%.

Hence, the patient’s treatment course began with Ruconest and Firazyr (subcutaneous bradykinin B2 receptor antagonists) as on-demand therapy and Orladeyo (an oral kallikrein inhibitor) for long-term prophylaxis (LTP). This treatment regimen reduced symptoms for a few days, after which she returned to the hospital for a severe laryngeal swell and was admitted to the ICU. Due to a lack of on-demand HAE medication availability, she was intubated and given glucocorticoids, Pepcid (an H2 antagonist), Benadryl (an H1 antagonist), Berinert (an intravenous plasma-derived C1 esterase inhibitor), and fresh frozen plasma (FFP).

After discharge, she was prescribed Haegarda (a subcutaneous plasma-derived C1 esterase inhibitor) for LTP, in addition to the Orladeyo. The patient continued to have episodes of angioedema, leading to several emergency department visits. Orladeyo and Firazyr were discontinued. Takhzyro (a subcutaneous kallikrein inhibitor) was added for LTP, and Sajazir (a subcutaneous bradykinin B2 receptor antagonist) was added for on-demand therapy. The patient continued these treatments for several months. Although the severity of the symptoms had attenuated, the patient continued to have episodes of significant angioedema, including one additional hospitalization.

The role of estrogen has been well documented as a factor in HAE nC1-INH exacerbations. The patient is now on a trial of Danazol, an anabolic androgen. She also consulted with an OB-GYN about a possible oophorectomy.

The patient’s quality of life has been severely affected, and a short-term disability has been obtained.

## Discussion

The patient has suffered from angioedema since adolescence. Recent analysis has shown 56.7% of patients with HAE nC1-INH experience a delay in diagnosis of at least 10 years from the initial presentation [8]. Due to the low prevalence of HAE nC1-INH, it is recommended that when patients are negative for HAE types 1 or 2, the diagnosis of HAE nC1-INH be entertained.

The mechanism through which HAE nC1-INH occurs is still being elucidated and does require further research. However, it has been hypothesized that HAE nC1-INH, like HAE C1-INH, may be bradykinin-mediated, as patients with either diagnosis may respond to bradykinin pathway-targeted medications [9]. This shows the need for further research into the drivers of HAE nC1-INH so that we may possibly reduce exacerbations and improve future patients’ quality of life.

Many cases of HAE nC1-INH have been shown to have a mutation in factor XII [10]. However, in this case, testing for this specific mutation came back negative. HAE nC1-INH has long been thought to have an estrogen-related component in addition to genetic mutations. High estrogen states, such as contraceptives and pregnancy, have been linked with increased severity and frequency of flares in HAE [10-11]. Increased estrogen has also been shown to exacerbate the disease process in 91% of female patients with HAE nC1-INH with a factor XII mutation [12]. This is likely due to estrogen's ability to upregulate the kallikrein-bradykinin system [2]. Due to the initial onset of the patient’s symptoms in adolescence, there is reason to suspect an increased estrogen level as a possible trigger. Interestingly, HAE nC1-INH has been shown to manifest more frequently in early adulthood than HAE C1-INH [12].

Curiously, our patient’s symptoms worsened approximately two years ago after an anaphylactic reaction to an initial dose of Spikevax. It is worth acknowledging that rarely, angioedema attacks have been noted post-vaccination in persons with a diagnosis of HAE as well as being implicated in an acquired case of angioedema [13-14]. However, other studies have asserted that mRNA vaccines are safe for administration in a variety of immediate-response hypersensitivity diseases, including HAE [13,15]. Hence, any link between angioedema in patients with HAE nC1-INH and COVID-19 vaccination is entirely speculative. It may warrant further investigation.

Perhaps the most important aspect of this patient’s case is her worsening symptoms, which could not be adequately controlled through LTP. The response to LTP in HAE nC1-INH is suboptimal and warrants further investigation [7]. This patient continued to have attacks despite being treated with a variety of LTP medications. Thankfully, the patient responds to Ruconest as an on-demand therapy.

As mentioned above, HAE nC1-INH was originally thought to be largely estrogen-linked so there is promise in combating her symptoms with danazol and a potential oophorectomy. However, due to the large side effect profile, these treatments are not currently considered first-line therapy [7]. These treatments need further investigation to address the estrogen-driven component of this disease.

## Conclusions

This case illustrates the many gaps in knowledge that we still have regarding HAE nC1-INH. There appears to be an underlying pathway and/or genetic mutations contributing to these episodes, and exploring these case studies is a necessary avenue to help elucidate the drivers of this disease. The life-threatening sequelae and decreased quality of life illustrate the need for further investigation in this population.
